# Fraxinol attenuates LPS-induced acute lung injury by equilibrating ACE-Ang II-AT1R and ACE2-Ang (1-7)-Mas and inhibiting NLRP3

**DOI:** 10.1080/13880209.2022.2067571

**Published:** 2022-05-19

**Authors:** Yan Wu, Xin Yang, Yuanyuan Ju, Fei Zhao

**Affiliations:** Department of Pediatrics, The Second Hospital, Cheeloo College of Medicine, Shandong University, Jinan, Shandong, China

**Keywords:** Inflammation, apoptosis, renin-angiotensin system

## Abstract

**Context:**

Acute lung injury (ALI) is a serious heterogenous pulmonary disorder. Fraxinol was selected for this study since it is a simple coumarin compound, not previously investigated in ALI.

**Objectives:**

This study investigates the ALI therapeutic effect and mechanisms of fraxinol.

**Materials and methods:**

Male BALB/c mice were treated with fraxinol (20, 40, and 80 mg/kg) following intranasal injection of lipopolysaccharide (LPS; 10 μg in 50 μL). The mice in control group were intratracheally injected with 50 μL phosphate buffered saline (PBS). Raw264.7 cells were treated with fraxinol by 100 ng/mL LPS for 6 h, then treated by different concentrations of fraxinol (5, 10, and 25 μM) for 48 h. Cells in control group were treated with PBS.

**Results:**

Fraxinol with doses of 20, 40, and 80 mg/kg significantly attenuated LPS-induced lung injury in mice (lung injury score, 10.4, 31.2, 50.3%). Fraxinol attenuated the apoptosis and nucleotide-binding oligomerization domain-like receptor family pyrin domain-containing-3 (NLRP3) activation induced by LPS (apoptosis, 18.3, 30.2, 55.6%; NLRP3, 30.0, 47.7, 63.6%). The anti-apoptosis and anti-inflammation effects of fraxinol were also confirmed in Raw264.7 cells (apoptosis, 38.8, 55.3, 68.9%; NLRP3, 20.6, 55.7, 73.9%).

**Discussion and conclusion:**

The anti-ALI effects of fraxinol maybe by equilibrating ACE-Ang II-AT1R and ACE2-Ang (1-7)-Mas axis and inhibiting NLRP3 inflammasome. Our research provides a candidate drug in the treatment of ALI.

## Introduction

Acute lung injury (ALI) is a serious heterogenous pulmonary disorder characterized by pulmonary edoema, diminished lung compliance, and the imbalance of ventilation/blood flow ratio (Trepte et al. 2015). The clinical manifestations include progressive hypoxaemia and respiratory distress (Saguil and Fargo 2020). The lung imaging manifests as non-uniform exudative lesions, and further develops into acute respiratory distress syndrome (ARDS) (Tomashefski 2000). Controlling the progression of ALI has been considered to be effective in preventing ARDS (Dushianthan et al. 2011). However, the treatment of ALI has not made significant progress, and the morbidity and mortality rates remain high (Schmickl et al. 2015).

Lipopolysaccharide (LPS), a main component of endotoxin, is the most important and widely used method for inducing lung injury in animals (Chen et al. 2010). LPS-induced rat models are able to mimic the typical features of lung injury detected in human, including lung tissue inflammation and pulmonary edoema (Niu et al. 2017). Under the stimulation of LPS, various inflammatory cytokines are secreted, such as interleukin (IL)-1β and tumour necrosis factor (TNF)-α, and they participate in immune responses via interaction with a variety of signalling pathways (Hu et al. 2017). In addition, there is increasing interest in investigation of Nod-like receptor protein 3 (NLRP3) inflammasome, as its critical roles in the secretion of cytokines (Zhang et al. 2017; 2017b).

Renin-angiotensin system (RAS) is a physiological regulation system that plays an important role in blood pressure regulation as well as water and electrolyte balance in the body (Li et al. 2012). As one of the main members of RAS, Angiotensin II (Ang II) is able to induce the occurrence and development of ALI/ARDS through a variety of ways when RAS is overactivated (Gao et al. 2020). Angiotensin converting enzyme (ACE)/Ang II/angiotensin type-1 receptor (AT1R) axis promotes RAS vascular constriction and inflammation (Saguil and Fargo 2020). Angiotensin-converting enzyme 2 (ACE2) is a multi-substrate metalloprotease that has been revealed to be a necessary functional receptor for severe acute respiratory syndrome (SARS) coronavirus infection (C et al. 2020; Lai et al. 2020). ACE2 plays a negative regulatory role in RAS by hydrolyzing Ang I and Ang II, and finally converting to Ang (1-7). Furthermore, Ang (1-7) act as endogenous Ang II antagonists which mediated by Mas receptor (MasR) with main functions of blood vessel relaxations, and heart and vascular endothelium improvement (Chen et al. 2018; Liu et al. 2018). Recent studies have suggested that regulation of ACE/Ang II/AT1R and its counter regulatory ACE2-Ang (1-7)-Mas axis has potential in treating ALI/ARDS (Kuba et al. 2005; Zhang et al. 2018; Wang et al. 2021).

Coumarin compounds are lactones of *O*-hydroxycinnamic acid with aromatic odour (Reen et al. 2018). Coumarins contain three main types: simple coumarins, furanocoumarins and pyranocoumarins (Bhattarai et al. 2021). They are all active ingredients in crude drugs that widely distributed in the plant kingdom, and have various biological activities such as antiradiation, antioxidation, antimicrobial, and antihypertensive (Annunziata et al. 2020). A growing number of coumarins have been suggested to be beneficial in preventing or treating ALI. For example, treating septic mice with osthole significantly inhibits the lung injury, leukocytic recruitment, and cytokine productions (Jin et al. 2018). Esculetin, a coumarin derivative, attenuates LPS-induced ALI in mice (Lee et al. 2020). More interestingly, coumarins exert theirs protective functions in lung possibly via modulating ACE2-Ang (1-7)-Mas axis (Shi et al. 2013; Hao and Liu 2016). In this study, Traditional Chinese Medicine Systems Pharmacology Database (TCMSP) and Traditional Chinese Medicine Systems Pharmacology Database (CTD) databases were used to predict the effective chemical components which are related with ALI. Four common effective chemical ingredients were obtained, coumarin, kaempferol, morin, and fraxinol. Fraxinol was selected for this study since it is a simple coumarin compound (Hammoda et al. 2008), not previously investigated in ALI. Both animal and cell models of ALI were established. The therapeutic effects of fraxinol on ALI were explored by testing lung injury, cytokines secretion, ACE/Ang II/AT1R, ACE2-Ang (1-7)-Mas regulation network and NLRP3 activation. The findings of this study provide experimental basis for ALI treatment.

## Materials and methods

### Online analysis tools

The effective chemical components corresponding to ALI were predicted in TCMSP (https://tcmsp-e.com/) by entering ‘inflammatory lung disease’ as the keyword. The CTD (http://ctdbase.org/) was used to predict the effective chemical components corresponding to the disease by entering ‘pneumonia’ as the keyword. The target proteins corresponding to pneumonia was predicted by CTD, malacards (https://www.malacards.org/MalaCards) and disgenet (https://www.disgenet.org/) databases. Swiss Target Prediction (http://www.swisstargetprediction.ch/) was used to predict the target proteins corresponding to fraxinol.

### Animals

Male BALB/c mice (22 ∼ 25 g) were provided by Shandong Provincial Laboratory Animal Centre (Shandong, China). The mice were randomly divided into 5 groups (6 mice per group): Control, LPS, LPS + (20 mg/kg) Fraxinol, LPS + Fraxinol (40 mg/kg), and LPS + Fraxinol (80 mg/kg). The mice in control group were intratracheally injected with 50 μL PBS. LPS group of mice were intratracheally injected with 10 μg of LPS in 50 μL PBS. LPS+ (20 mg/kg) Fraxinol, LPS + Fraxinol (40 mg/kg), and LPS + Fraxinol (80 mg/kg) groups were injected with 20, 40, or 80 mg/kg fraxinol into the intraperitoneal cavity following LPS stimulation. LPS from *Escherichia coli* O111:B4 was purchased from Sigma-Aldrich (St. Louis, MO, USA). Fraxinol was obtained from MedChemExpress (Monmouth Junction, NJ, USA). After 12 h of treatment in the above groups, the bronchoalveolar lavage fluid (BALF) and lung tissues of mice were collected following sacrifice. The experimental protocol of our study was performed in accordance with the Guide for the Care and Use of Laboratory Animals and approved by the Second Hospital, Cheeloo College of Medicine, Shandong University (No. SDDDW20200405).

### Haematoxylin and eosin (H&E) staining

The middle lobe of the right lung was taken, fixed with 4% paraformaldehyde, dried in alcohol, embedded in paraffin, and the sections were stained with H&E and observed under an optical microscope. Lung injury score was measured according to the methods reported previously (Wang et al. 2021). The criteria are as follows: score 0 = no damage, score l = mild damage, score 2 = moderate damage, score 3 = severe damage, score 4 = very severe histologic changes.

### Lung wet/dry (W/D) ratio

After the mice were euthanized, the lung tissues were separated and weighed immediately. The lung tissue was dried in an oven at 80 °C for 72 h to reach a stable dry weight. The lung W/D ratio was measured to indicate the water content of the lung tissue.

### Myeloperoxidase (MPO) assay

After collecting and homogenizing the lungs, the MPO activity detection kit (Nanjing Jiancheng Institute of Bioengineering, Nanjing, China) was used to measure the MPO activity according to the manufacturer's instruction.

### TUNEL assay

The lung tissues were fixed in 4% formaldehyde, prepared into paraffin specimens, and sliced. After the sections were completely deparaffinized and hydrated, the sections were added freshly prepared 3% H_2_O_2_ deionized water and covered the section specimens, and incubated at room temperature for 15 min to block endogenous peroxidase activity. The sections were incubated with proteinase K working solution at 37 °C for 20 min, and appropriate amount of reagent 1 (TdT) and reagent 2 (dUTP) was added dropwise to the slices. After the reaction was terminated with clear water, the nuclei were stained with haematoxylin for 10 min and observed under an optical microscope.

### Cell culture

Raw264.7 cells were purchased from American Type Culture Collection (Manassas, VA, USA) and cultured in 90% Dulbecco’s Modified Eagle’s Medium (Gibco, Grand Island, NY, USA) with 10% foetal bovine serum (Hyclone, Logan, UT, USA). The culture conditions were 95% air, 5% CO_2_ at 37 °C. For inducing a cell model of ALI, Raw264.7 cells were treated by 100 ng/mL LPS for 6 h. The cells were then treated by different concentrations of fraxinol (5, 10, and 25 μM) for 48 h. The cells were cultured with 10^−5 ^mol/L A779 (MedChemExpress) and 10^−5 ^mol/L MLN-4760 (MedChemExpress) for inhibition of MasR and ACE2. Nigericin with concentration of 10 µM was used to treat cells for activation of NLRP3.

### Enzyme-linked immunosorbent assay (ELISA)

After preparing the coating solution, 0.1 mL of a certain diluted sample was added to coated reaction well at 37 °C for 1 h strictly following the manufacturer's instruction. Then, 0.1 mL freshly diluted enzyme-labeled antibody was added and incubated at 37 °C for 1 h, then substrate solution for colour development was incubated for 30 min and stop solution was added. The level of TNF-α, IL-1β, Ang II, and Ang (1-7) was detected using ELISA detection kits (R&D Systems, UK). ELISA assay was performed in triplicate.

### Flow cytometry

The apoptosis of Raw264.7 cells was detected by using Annexin V-PE Apoptosis Detection Kit (Beyotime, Shanghai, China). Following treatment, 1 × 10^5^ cells per sample was collected and resuspended in 195 μL Annexin V-PE binding buffer. Cells were then stained with 5 μL Annexin V-PE for 20 min at room temperature in the dark. Apoptosis cell number was calculated by a flow cytometry (BD Biosciences, San Jose, CA, USA).

### Western blot assay

The expression of ACE, AT1R, ACE2, MasR, NLRP3, ASC, pro-caspase-1, cleaved caspase-1, Na/K-ATPase and β-actin in lung tissues and Raw264.7 cells were detected by Western blotting. Briefly, RIPA lysate (CWBIO, Beijing, China) was used to extract the whole proteins in tissues and cells. Membrane Protein Extraction Kit (Sangon Biotech, Shanghai, China) was used for extraction of membrane protein. The protein analyses were carried out by sodium dodecyl sulfate-polyacrylamide gel electrophoresis, transferring membrane, and antibody incubation. Image J software (IPP, TX, USA) was used for semi-quantitative analysis. ACE (1:1000, cat. no. K003493M, Solarbio, China), AT1R (1:5000, cat. no. ab124734, Abcam, USA), ACE2 (1:5000, cat. no. ab108252, Abcam, USA), MasR (1:1000, cat. no. ab200685, Abcam, USA), NLRP3 (1:1000, cat. no. K008087P, Solarbio, China), ASC (1:1000, cat. no. #67824, Cell Signalling Technology, USA), pro-caspase-1 (1:1000, cat. no. ab179515, Abcam, USA), cleaved caspase-1 (1:1000, cat. no. #89332, Cell Signalling Technology, USA), Na/K-ATPase (1:1000, cat. no. #3010, Cell Signalling Technology, USA), β-actin (1:2000, cat. no. K101527P, Solarbio, China), and Goat Anti-rabbit IgG/HRP antibody (1:5000, cat. no. SE134, Solarbio, China).

### Statistical analysis

SPSS18.0 software was used for statistical analysis. The data of each group were expressed as mean ± standard deviation. The difference between groups was analyzed by one-way analysis of variance or independent sample *t*-test. *p* < 0.05 was considered statistically significant.

## Results

### Network pharmacology analysis of Chinese medicine to pneumonia

**Figure 1.**
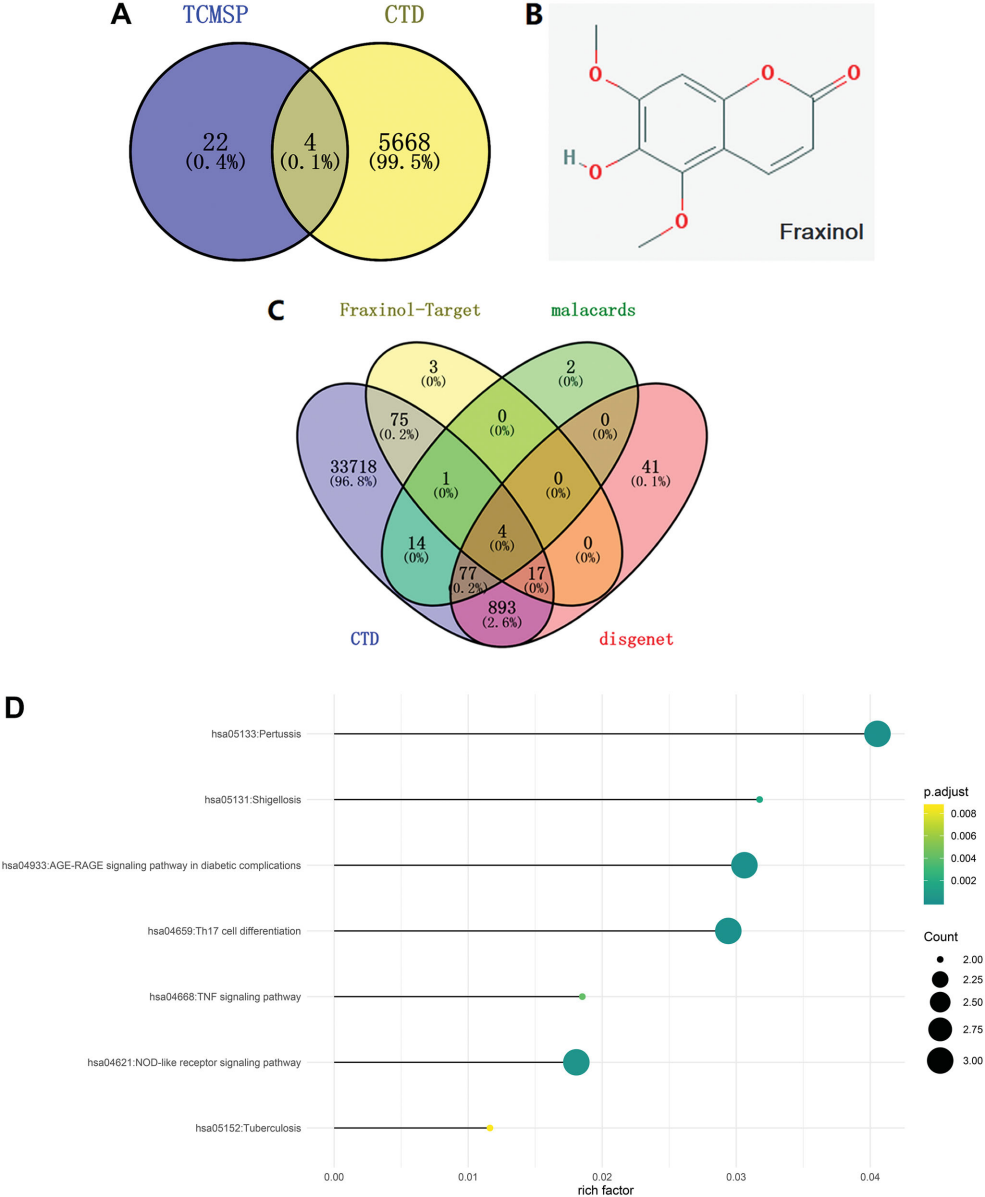
Network pharmacology analysis of Chinese medicine to pneumonia. (A) The effective chemical components corresponding to inflammation lung disease are predicted through TCMSP and CTD database. (B) The structure of fraxinol. (C) The target proteins corresponding to pneumonia and fraxinol are predicted by CTD, malacards, disgenet, and Swiss Target Prediction databases, respectively. (D) The related signalling pathways were predicted through overlapping analysis of the pneumonia signalling and target proteins.

The effective chemical components corresponding to the inflammation lung disease were predicted through TCMSP database, and 26 effective chemical components were obtained. Also, 5672 kinds of effective chemical components were screened out by using CTD database. There are four overlapped active ingredients, including fraxinol, coumarin, kaempferol, and morin (Figure 1(A)). Considering fraxinol has not been studied in ALI, it was selected for use in the following studies. The molecular structure of fraxinol was shown in Figure 1(B). The target proteins corresponding to pneumonia were then predicted by searching on CTD, malacards, and disgenet databases. Meanwhile, the target proteins corresponding to fraxinol were screened out by using Swiss Target Prediction database. As shown in Figure 1(C), the overlapped proteins were as following: ACE, MAPK8, F2, and ELANE. ACE was selected for the further studies. Moreover, the downstream signalling pathways were screened out by the overlapping analysis. As shown in Figure 1(D), NLRP3 inflammasome was predicted as the most relevant signalling pathway, which was selected for the further studies.

### Fraxinol attenuates LPS-induced ALI in mice

**Figure 2.**
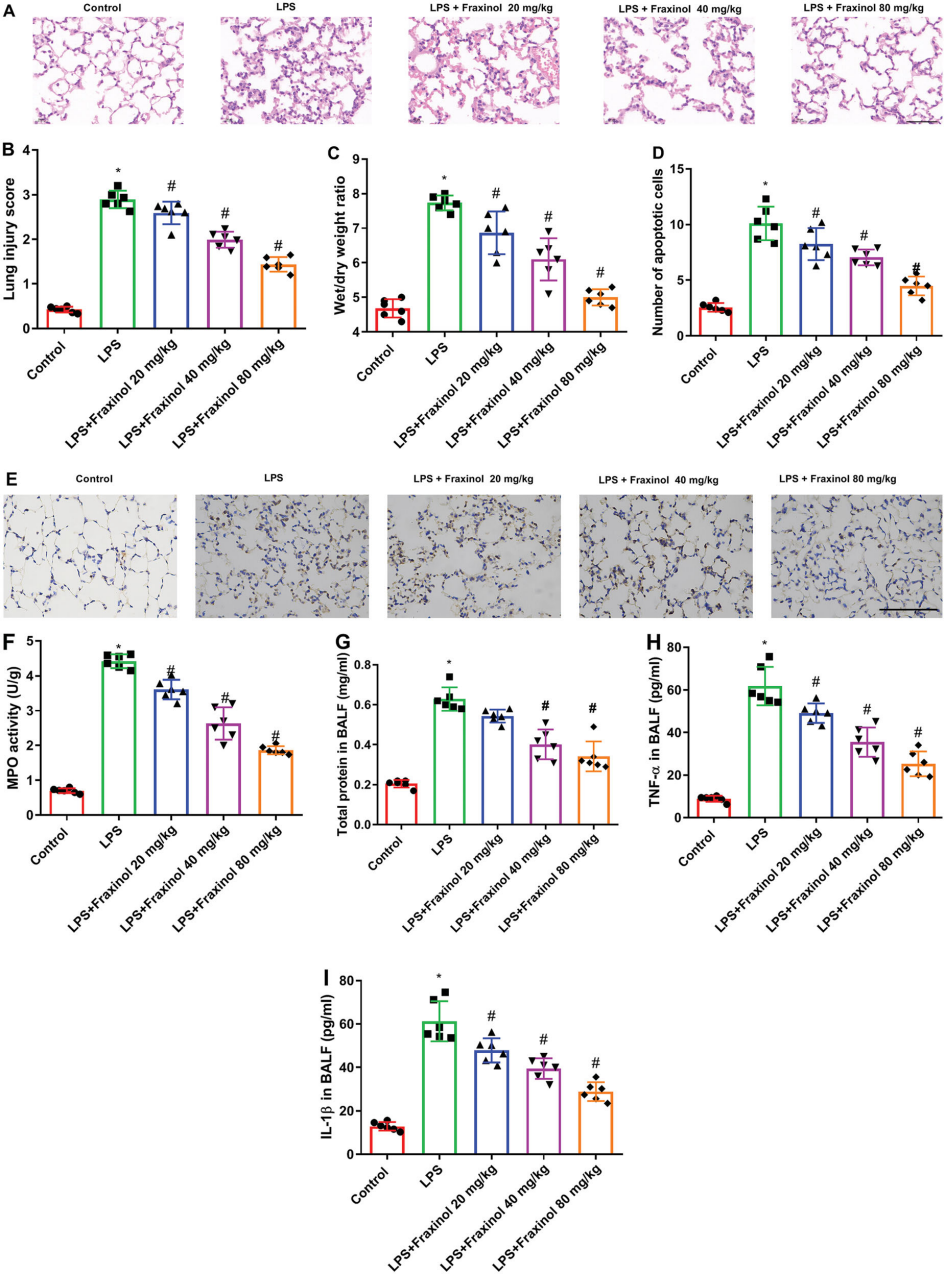
Fraxinol attenuates LPS-induced ALI in mice. **(**A,B) Histological changes were evaluated by using H&E staining. Bar = 20 μm. (C) The lung W/D ratio was used to assess the pulmonary edoema. (D,E) Apoptotic cells were measured by TUNEL assay. Bar = 20 μm. (F) The level of MPO activity. (G) Effect of fraxinol on total proteins in BALF. (H,I) TNF-α and IL-1β levels in BALF were measured by ELISA kits. *p < 0.05 vs. Control group; ^#^*p* < 0.05 vs. LPS group.

To verify the effects of fraxinol on ALI, a mouse model of ALI was established by using LPS. The histological changes were evaluated by H&E staining. The result in Figure 2(A,B) showed that LPS caused obvious pathological changes as compared to the control group, including observable inflammatory cell infiltration, haemorrhage and alveolar wall thickening, and capillary congestion. On the contrary, in the LPS + Fraxinol groups, these changes and lung structural destruction were significantly improved.

Then the lung W/D ratio was calculated to observe the pulmonary edoema. The result in Figure 2(C) showed that, the lung W/D ratio of the LPS group was significantly increased as compared with the control group, while fraxinol significantly inhibited the ratio. In Figure 2(D,E), the number of apoptotic cells was increased after LPS stimulation, whereas fraxinol treatment inhibited the increase in the number of apoptotic cells in LPS-induced mice. As shown in Figure 2(F), LPS resulted in a marked increase in the level of MPO activity as relative to the control group. However, fraxinol dramatically relieved LPS-induced MPO activity. As illustrated in Figure 2(G–I), LPS stimulation caused significantly increases in total protein, and TNF-α and IL-1β levels in BALF. The increases of total protein and cytokine levels were dramatically reduced by fraxinol treatment.

### Fraxinol alleviates LPS-induced ALI by equilibrating ACE-Ang II-AT1R and ACE2-Ang (1-7)-mas in mice

**Figure 3.**
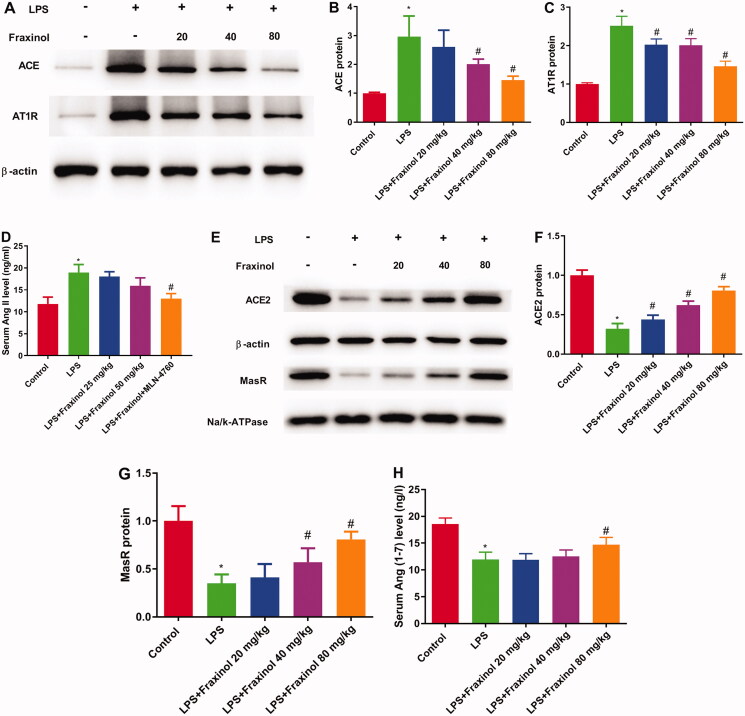
Fraxinol alleviates LPS-induced ALI by equilibrating ACE-Ang II-AT1R and ACE2-Ang (1-7)-Mas in mice. (A–C) The protein expression of ACE and AT1R was measured using Western blot assay. (D) The level of Ang II was detected by ELISA kit. (E–G) The protein expression of ACE2 and MasR was measured using Western blot assay. (H) The level of Ang (1–7) was detected by ELISA kit. *p < 0.05 vs. control group; ^#^*p* < 0.05 vs. LPS group.

The result in Figure 3(A–D) showed that the levels of ACE, Ang II, and AT1R in the LPS group was significantly increased as compared with the control group, while fraxinol significantly inhibited the levels of ACE, Ang II, and AT1R. As shown in Figure 3(E–H), ACE2, Ang (1-7) and MasR levels in the LPS group were remarkably decreased compared with the control group and significantly increased by fraxinol treatment. These data suggest that fraxinol alleviates LPS-induced ALI by equilibrating ACE-Ang II-AT1R and ACE2-Ang (1-7)-Mas.

### Fraxinol ameliorates LPS-induced NLRP3 inflammasome in mice

**Figure 4.**
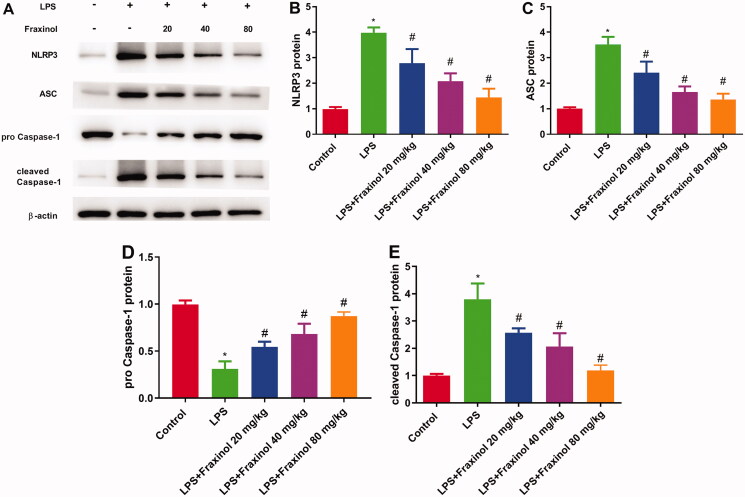
Fraxinol ameliorates LPS-induced NLRP3 inflammasome in mice. (A–E) The protein expression of NLRP3, ASC, pro-caspase-1, and cleaved caspase-1 was measured using Western blot assay. *p < 0.05 vs. Control group; ^#^*p* < 0.05 vs. LPS group.

To evaluate the anti-inflammatory mechanism of fraxinol, the activation of NLRP3 signalling pathway was detected. LPS administration led to increased expression of NLRP3, ASC, and cleaved caspase-1, while decreased expression of pro-caspase-1. These effects were significantly blunted by fraxinol treatment in LPS-induced ALI mice (Figure 4(A–E)). These results suggest that fraxinol attenuates LPS-induced ALI by inhibiting the NLRP3 signalling pathway.

### Fraxinol ameliorates LPS-induced Raw264.7 cell inflammation and apoptosis via equilibrating ACE-AngII-AT1R and ACE2-Ang (1-7)-mas

**Figure 5.**
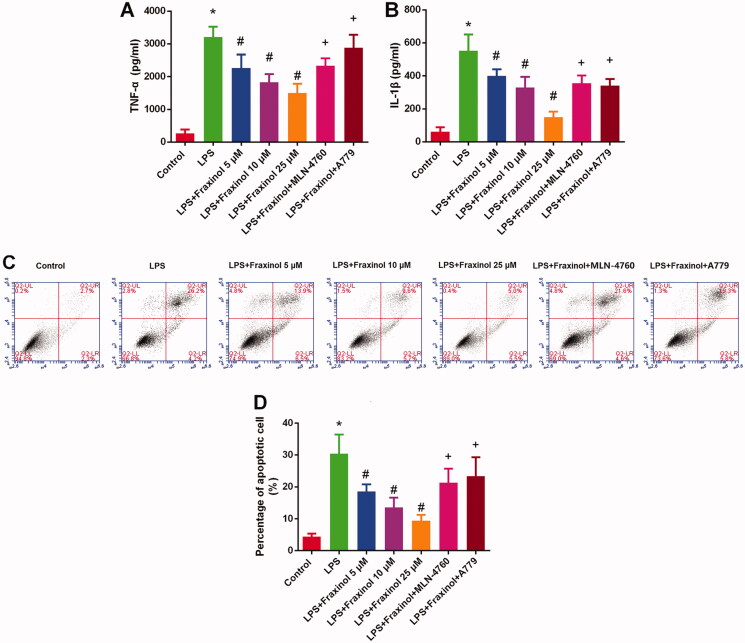
Fraxinol ameliorates LPS-induced Raw264.7 cell inflammation and apoptosis. (A,B) Effects of fraxinol on LPS-induced TNF-α and IL-1β levels in Raw264.7 cells were detected by ELISA kits. (C,D) Effects of fraxinol on LPS-induced apoptosis in Raw264.7 cells were detected by flow cytometry. *p < 0.05 vs. Control group; ^#^*p* < 0.05 vs. LPS group; ^+^*p* < 0.05 vs. LPS + Fraxinol (25 μM) group.

**Figure 6.**
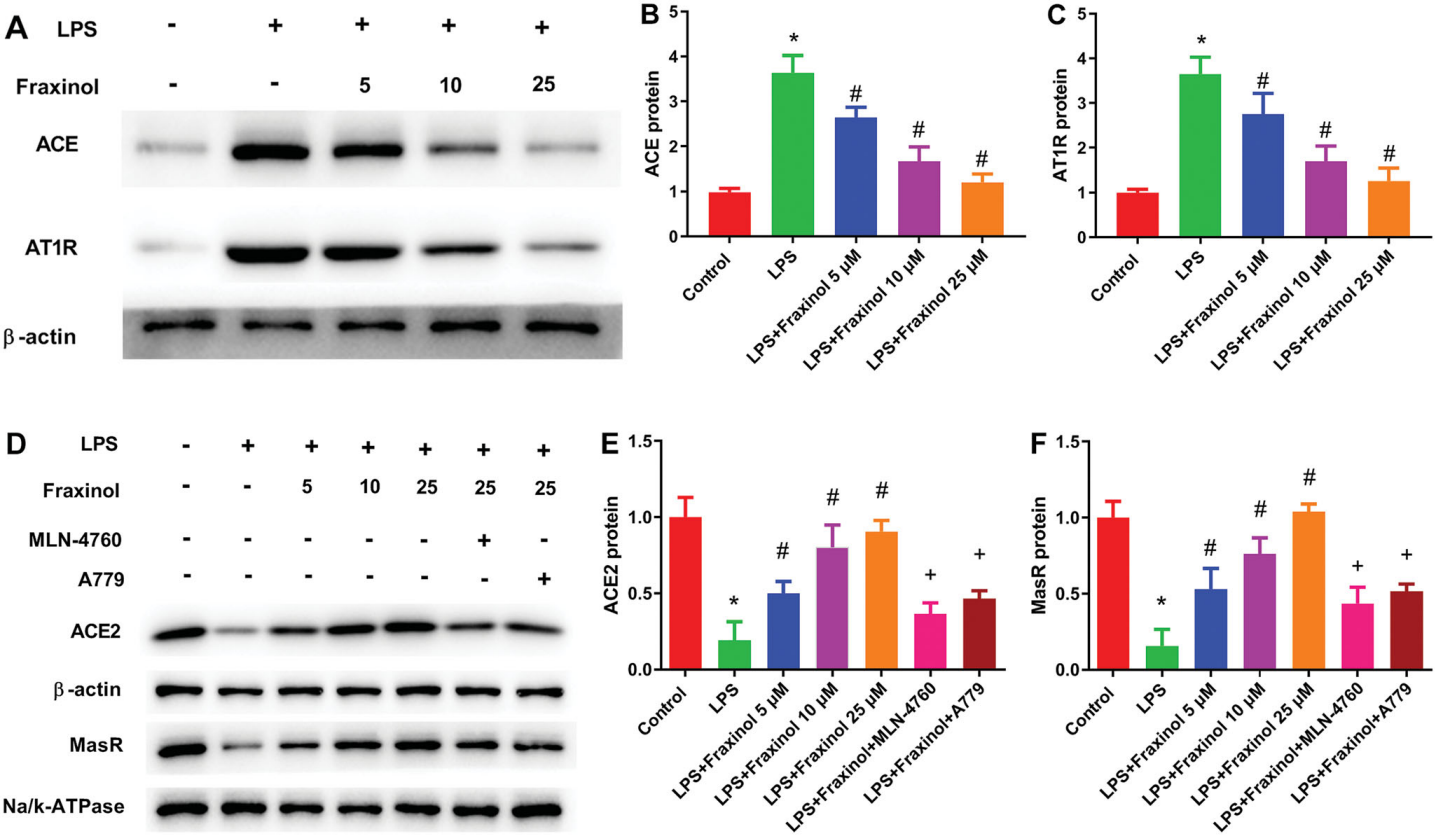
Fraxinol equilibrates ACE-Ang II-AT1R and ACE2-Ang (1-7)-Mas axis in Raw264.7 cells. (A–C) The protein expression of ACE and AT1R was measured by Western blot assay. (D–F) The protein expression of ACE2 and MasR was measured by Western blot assay. *p < 0.05 vs. Control group; ^#^*p* < 0.05 vs. LPS group; ^+^*p* < 0.05 vs. LPS + Fraxinol (25 μM) group.

Raw264.7 cells were treated with LPS, and then the functional effects of fraxinol were studied *in vitro*. As results shown in Figure 5(A,B), concentrations of TNF-α and IL-1β in cell supernatant were dramatically increased by LPS treatment. Fraxinol attenuated the LPS-induced TNF-α and IL-1β levels in a dose-dependent manner. Besides, MLN-4760 and A779 were effective in reversing the effects of fraxinol on TNF-α and IL-1β levels. LPS induced a significant increase in cell apoptosis (Figure 5(C,D)). Fraxinol inhibited apoptosis in a dose-dependent manner, while MLN-4760 and A779 promoted the apoptosis. In addition, the protein expression of ACE and AT1R was upregulated by LPS (Figure 6(A–C)), while the protein expression of ACE2 and MasR was downregulated by LPS (Figure 6(D–F)). Fraxinol attenuated the effects of LPS on these proteins, and the effects of fraxinol were reversed by MLN-4760 and A779. These data confirm the protective function of fraxinol and its mechanism *in vitro*.

### Fraxinol ameliorates LPS-induced Raw264.7 cell inflammation and apoptosis via regulating NLRP3

**Figure 7.**
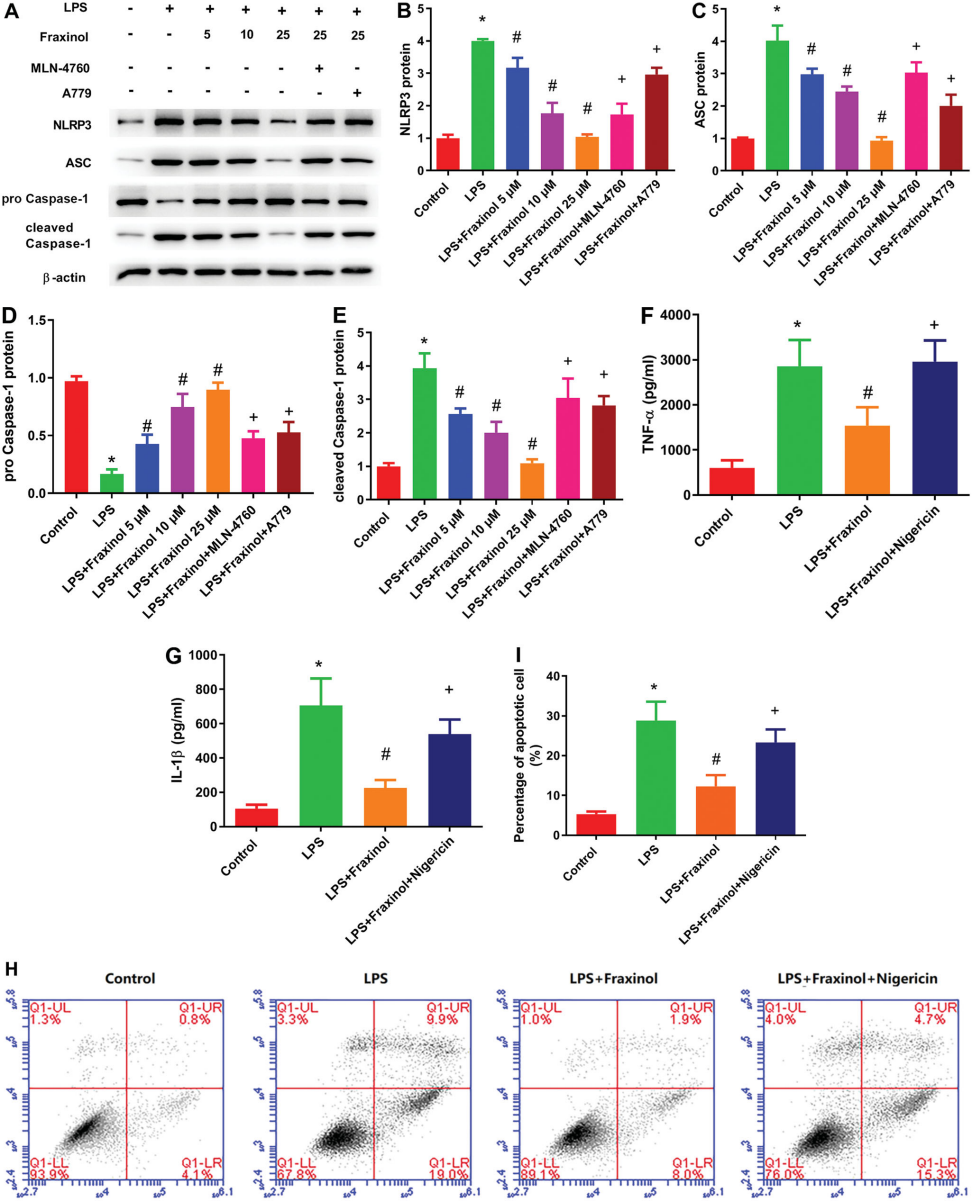
Fraxinol inhibits LPS-induced Raw264.7 cell inflammation and apoptosis via regulation of NLRP3. (A–E) The protein expression of NLRP3, ASC, pro-caspase-1, and cleaved caspase-1 was measured by Western blot assay. (F,G) Effects of fraxinol and nigericin on LPS-induced TNF-α and IL-1β levels in Raw264.7 cells were detected by ELISA kits. (H,I) Effects of fraxinol and nigericin on LPS-induced apoptosis in Raw264.7 cells were detected by flow cytometry. *p < 0.05 vs. Control group; ^#^*p* < 0.05 vs. LPS group; ^+^*p* < 0.05 vs. LPS + Fraxinol (25 μM) group.

To confirm whether fraxinol inhibits LPS-induced inflammation and apoptosis in Raw264.7 cells via regulation of NLRP3, an agonist of NLRP3 (nigericin) was used. The protein expression of NLRP3, ASC, and cleaved caspase-1 was upregulated by LPS, while the protein expression of pro-caspase-1 was downregulated by LPS (Figure 7(A–E)). Fraxinol attenuated the effects of LPS on these proteins, and the effects of fraxinol were reversed by MLN-4760 and A779. As shown in Figure 7(F,G), the inhibitory effects of fraxinol on TNF-α and IL-1β levels were significantly attenuated by treating Raw264.7 cells with nigericin (*p* < 0.05). Additionally, the inhibitory effects of fraxinol on cell apoptosis were attenuated by nigericin (Figure 7(H,I)). These data suggest that fraxinol ameliorates LPS-induced Raw264.7 cell inflammation and apoptosis at least in part via regulation of NLRP3.

## Discussion

ALI is characterized by uncontrolled cytokine-mediated lung inflammation (Tomashefski 2000). NLRP3 inflammasome plays an important role in regulating the process of immune inflammatory response and disease occurrence (Zhang et al. 2017a). NLRP3 inflammasome can regulate the activation of caspase-1 and promote the secretion of various inflammatory factors, such as IL-1β and IL-18. Inhibiting the activation of NLRP3 inflammasome has been considered as a new therapeutic target of various inflammatory diseases, including ALI (Zhang et al. 2016; Sun et al. 2019). For example, andrographolide has been reported to be effective in inhibiting LPS-induced lung injury in mice through regulation of NLRP3 activation (Segovia et al. 2018;; Lecoeur et al. 2020). In this study, fraxinol exerted similar effects in inhibiting NLRP3 activation. Meanwhile, experimental results showed that fraxinol reduced the secretion of inflammatory factors TNF-α and IL-1β, and downregulated the expression of NLRP3, ASC, and cleaved caspase-1. A growing number of studies have shown that NLRP3 inflammasome is one of the important signals for the production of inflammatory cytokines (Grebe et al. 2018; Mao et al. 2018; Sendler et al. 2020). The activation of NLRP3 inflammasome induces the secretion of pro-inflammatory cytokines, such as IL-1β and IL-18. Once activated, NLRP3 binds to ASC and causes caspase-1 cleavage, which further induces the secretion and maturation of IL-1β and IL-18 (Jin et al. 2020). Our data suggest that fraxinol exerts its anti-inflammatory effects on LPS-induced Raw264.7 cells possibly via regulating NLRP3 activation.

ACE-Ang II-AT1R axis promotes the process of ALI, whereas the ACE2-Ang-(1-7)-Mas axis inhibits it (Xu et al. 2017). In the current study, fraxinol was found to be effective in downregulating ACE and AT1R proteins and upregulating ACE2 and MasR proteins. The anti-ALI effects of fraxinol can be abolished by using ACE2 inhibitor (MLN-4760) and MasR inhibitor (A779). In addition, it has been reported that ACE2-Ang (1-7)-Mas inhibits the activation of NLRP3 inflammasome (You et al. 2019; Huang et al. 2020; Dang et al. 2021). This was also confirmed in this study, as inhibition of ACE2 and MasR significantly increased NLRP3 activation.

## Conclusions

Fraxinol attenuates LPS-induced ALI by inhibiting the production of inflammatory cytokines and NLRP3 activation. The mechanism behind the protection is related to the regulation of ACE-Ang II-AT1R and ACE2-Ang (1-7)-Mas. These findings provide new insight in regard to using fraxinol as a potential therapeutic drug to prevent LPS-induced ALI.

## Data Availability

The datasets used and analysed during the current study are available from the corresponding author on reasonable request.
